# Fibrin-derived peptide Bβ15-42 (FX06) as salvage treatment in critically ill patients with COVID-19-associated acute respiratory distress syndrome

**DOI:** 10.1186/s13054-020-03293-8

**Published:** 2020-09-24

**Authors:** Elisabeth H. Adam, Benedikt Schmid, Michael Sonntagbauer, Peter Kranke, Kai Zacharowski, Patrick Meybohm

**Affiliations:** 1 Department of Anaesthesiology, Intensive Care Medicine and Pain Therapy, University Hospital Frankfurt, Goethe-University, Frankfurt/Main, Germany; 2 Department of Anaesthesiology and Critical Care, University Hospital Wuerzburg, Julius-Maximilians-University, Wuerzburg, Germany

**Keywords:** Critical care, Respiratory distress syndrome, adult, COVID-19, Therapies, investigational, Pulmonary edema, Immunomodulatory agents

To the editor,

After SARS-CoV-2 first occurred in China in December of 2019, it set out to become a global pandemic. Critically ill patients constitute about 2–9% of all infected patients and progress from pneumonia and hypoxemia to multi-organ dysfunction, for which acute treatment options are scarce [1]. Currently, there is no clinical evidence supporting the efficacy and safety of a drug against any coronavirus in humans, including SARS-CoV-2. Here, we describe the empirical salvage treatment of critically ill COVID-19 patients in two German tertiary care University Hospitals with FX06 (F4 Pharma, Vienna, Austria), a naturally occurring peptide derived from the neo-N-terminus of fibrin (Bβ15-42). FX06 is known for its immunomodulatory properties [2] and was already investigated in clinical trials demonstrating convincing efficacy while being tolerated well with a favorable safety profile [3].

This observational case series includes six patients during their treatment in the intensive care unit. The respective institutions’ ethics committees approved the post hoc analysis of patient records for scientific purposes. The diagnosis of ARDS was based on the criteria put forth by the Berlin Definition.

Six mechanically ventilated patients suffering from moderate to severe ARDS upon ICU admission were treated with i.v. FX06 (400–600 mg per day; 3–7 days). Five out of these six patients additionally needed ECMO treatment during the course of their illness. Detailed clinical information is given in Table 1.

**Table 1:** Demographics and clinical characteristics at admission and treatment of patients

	Patient 1	Patient 2	Patient 3	Patient 4	Patient 5	Patient 6	
Age (Y)	52	78	63	51	71	55	
Sex	Male	Male	Male	Female	Male	Male	
BMI	31	35	26	54	28	37	
Comorbidities	Obesity	Obesity, coronary artery disease, arterial hypertension	Bronchial asthma	Obesity, arterial hypertension, rheumatoid arthritis	Type 2 diabetes mellitus	Obesity, arterial hypertension	
Invasive ventilation	Yes	Yes	Yes	Yes	Yes	Yes	
Severity of ARDS at admission	Moderate	Moderate	Moderate	Moderate	Severe	Moderate	
Anti-infective therapy	Imipeneme	Imipeneme	Imipeneme, voriconazol	Piperacillin/tazobactam, ciprofloxa-cin, meropenem, vancomycin, anidulafun-gin	Merope-neme, co-trimoxazol	Ampicillin/sulbactam, cephazolin, caspofungin	
Days on ICU prior to FX06 treatment	0	3	4	10	15	2	
SAPS II Score	57	75	43	68	63	59	
P_a_O_2_/F_i_O_2_ ratio at admission	186	141	131	154	85	122	
Daily dose of FX06	500 mg	600 mg	400 mg	400 mg	400 mg	400 mg	
Duration of FX06 treatment (days)	7	7	4	3	4	4	
vv-ECMO therapy	Yes	No	Yes	Yes	Yes	Yes	
Outcome	Rehabilitation care (after 35 days)	Death	Rehabilitation care (after 70 days)	Death	Rehabilitation care (after 48 days)	Rehabilitation care (after 44 days)	
Laboratory results at admission							Reference range
White blood cell count (cells per 10^6^/L)	14.02	15.56	6.26	7.9	14.2	11.2	3.92–9.81
Lymphocyte (cells per 10^6^/L)	1.12	1.24	0.71	0.92	1.44	1.32	1.05–3.24
Platelets	320	147	171	161	272	255	146–328
LDH U/L	378	1277	417	611	516	609	< 248
Creatinine mg/dL	0.72	2.34	0.43	0.50	0.82	0.88	0.7–1.2
C-reactive protein (mg/dL)	20.13	18.08	8.00	15.64	18.09	24.85	< 0.5
Ferritin ng/mL	883	5505	3708	1114	4079	3503 (day 3)	18–360
Procalcitonin ng/mL	0.15	0.30	0.78	0.09	1.32	2.44	< 0.5
Lactate mg/dL	9.0	14	9.0	8.1	12.6	13.5	4.5–14.5
IL-6 pg/mL	92.3	25.4	250	2647.0	440.9	360.1	< 7
D-dimer ng/mL	629	130,100	1056	450	2850	3750	< 500
aPTT (s)	28	30	29	48.6	44.0	37.8	25–37
vWF AG (%)	283	446	311	n/a	> 150	> 150	60–150

Demographics and clinical characteristics at admission and treatment of patients

*Y* years, *BMI* body mass index, *ARDS* acute respiratory distress syndrome, *SAPS* simplified acute physiology score, *LDH* lactate dehydrogenase, *U* units, *aPTT* activated partial Thromboplastin time, *VWF AG* von Willebrand factor antigen, *SAPS II* Simplified Acute Physiology Score, *P*_*a*_*O*_*2*_ partial pressure arterial oxygen, *F*_*i*_*O*_*2*_ fraction of inspired oxygen, *vv* veno-venous, *ECMO* extracorporeal membrane oxygenation

Mean oxygenation ratio improved over the first 3 days after the beginning of FX06 application, returned to baseline and increased steadily afterwards from day seven on (Fig. 1a). IL-6 serum concentrations as a marker of inflammation activity were instantly declining from day one (Fig. 1b). Norepinephrine dosages decreased initially after the initiation of FX06 therapy before returning to near-baseline values after some days (data not shown). Renal replacement therapy was necessary in four patients. Overall, four out of six patients survived. Both deceased patients (pats. 2 and 4 in Table 1) died from multi-organ failure due to septic shock most likely from secondary bacterial (co)infection. Hence, we saw no indication that the application of FX06 was in any way related to a patient’s death.

**Fig. 1:**
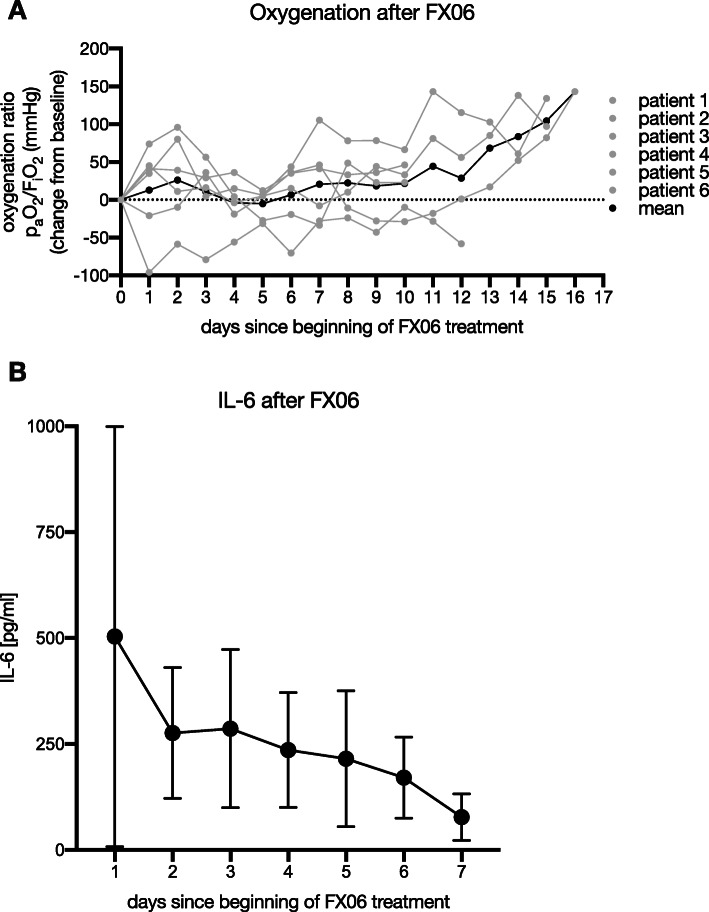
Oxygenation and IL-6 serum concentrations after FX06 treatment. **a** The difference in oxygenation compared to baseline (before FX06 treatment). p_a_O_2_^,^ partial pressure arterial oxygen; F_i_O_2_, fraction of inspired oxygen. **b** The course of interleukin 6 during the treatment with FX06. Data are presented as mean ± standard deviation

In summary, we observed substantial improvement in lung function following FX06 administration, which may be attributed to its immunomodulatory properties [3] and its function to preserve the endothelial barrier [4]. Patients treated with FX06 displayed a remarkable increase of their oxygenation indices, which we consider to be indicative of the normalization of the pulmonary vascular walls through the aforementioned underlying mechanisms. This was also mirrored in the radiographic diagnostics in five out of all six patients, reflecting a normalization of the interface between the alveolar space and an enhanced tissue integrity. Various coagulation factors, including fibrin degradation products, modulate the inflammatory response by influencing leukocyte migration and cytokine production [5, 6]. The decrease in IL-6 after FX06 is therefore considered to be attributed to these immunomodulatory effects.

Based on our experience, the salvage use of FX06 in severe COVID-19-associated ARDS could be an effective therapy to improve pulmonary function and vascular leakage in the most severely ill patients. A prospective randomized, controlled study to better elucidate this hypothesis is on preparation.

## Data Availability

The datasets used and/or analyzed during the current study are available from the corresponding author on reasonable request.

## Abbreviations

ARDS: Acute respiratory distress syndrome
SARS-CoV-2: Severe Acute Respiratory Syndrome Coronavirus 2
ICU: Intensive care unit
IL-6: Interleukin 6
ECMO: Extracorporeal membrane oxygenation
